# One year cumulative incidence and risk factors associated with workplace violence within the ambulance service in a Swedish region: a prospective cohort study

**DOI:** 10.1136/bmjopen-2023-074939

**Published:** 2024-09-05

**Authors:** Magnus Viking, Karin Hugelius, Erik Höglund, Lisa Kurland

**Affiliations:** 1 Department of Ambulance Care, Faculty of Medicine and Health, Örebro University, Örebro, Sweden; 2 Faculty of Medicine and Health, Örebro University, Örebro, Sweden

**Keywords:** health & safety, risk management, accident & emergency medicine

## Abstract

**Abstract:**

**Objective:**

To measure the 1 year cumulative incidence of and analyse the risk factors associated with workplace violence directed towards the ambulance service in a Swedish region.

**Design:**

Prospective cohort study.

**Setting:**

The ambulance services in Örebro County Council (Sweden) contain approximately 300 000 inhabitants.

**Participants:**

All ambulance missions during the period of 12 months (n=28 640) were assessed.

**Primary and secondary outcome measures:**

The primary outcome measure was workplace violence together with the associated risk factors.

**Results:**

The 1 year cumulative incidence of workplace violence within the ambulance service was 0.7%. Non-physical violence was most common. There was an increased odds for violence when the patient was under the influence of alcohol or drugs or suffering from mental illness. There was an association between the dispatch categories intoxication, unconsciousness or mental health problems and workplace violence against ambulance personnel. The offenders were mostly men aged 18–29 and workplace violence was more likely to occur in public places.

**Conclusions:**

The 1 year cumulative incidence of workplace violence within the regional ambulance service was low in comparison to that of previous research. The overall regression model had low explanatory power, indicating that the phenomenon is complex and that additional variables need to be taken into account when trying to predict when workplace violence will occur. Additional research is needed to fully understand why workplace violence within the ambulance service occurs and how to mitigate such situations.

**STRENGTHS AND LIMITATIONS OF THIS STUDY**

- Data was collected prospectively during 1 year.
- Data on risk factors was collected for all ambulance missions as part of the ambulance record.
- Data collection was performed in one Swedish region.
- The results indicate that there are variables other than those measured in the current study that influence workplace violence.

## Background

 Workplace violence is a worldwide problem within emergency care.^1^,^7^ Globally, there are different healthcare structures and ways to organise emergency care. In Sweden, the ambulance is the first responder to emergency calls. Hence, the current study focuses on workplace violence towards the ambulance service.^1 8^

It has previously been shown that ambulance personnel have the highest rate of occupational injury and fatality, and many of these injuries resulting from workplace violence.^9^,^12^ Workplace violence within the ambulance service has been reported to be between 0.8% and 5% of all ambulance missions,^13^,^15^ and some studies suggest that workplace violence is an increasing problem.^11 16^ Prior studies suggest that it is the patient who is violent and threatening,^717^,^19^ and a study from Sweden suggests that the violence most often occurs in the patient’s home.^8^ There are also prior studies suggesting that ambulance missions in the late afternoon and evening,^13^ patients with mental illness,^20^ young adults,^16^ men^21^ and patients under the influence of alcohol or drugs^8 15 22^ are risk factors for workplace violence. However, many of these prior studies are not recent and will therefore not reflect the changes in healthcare, socioeconomic and political changes. In addition, several studies had a retrospective study design. Thus, motivating the current prospective study of workplace violence in the ambulance.

Workplace violence can consist of physical violence, such as hitting, kicking, pushing or sexual abuse. Non-physical violence includes, for example, verbal abuse, threats, sexual harassment and bullying^23 24^ and has been shown to be more common than physical violence,^7 9 17 25^ motivating recording both physical and non-physical violence. Workplace violence directed towards the ambulance services can have severe consequences for the exposed ambulance personnel, causing psychological stress and an increase in burnout.^26^ On a societal level, workplace violence can affect ambulance personnel’s ability to provide safe and high-quality care.^1 8 27^ In order to enable a safe work environment for the ambulance personnel and safe care for the patient, there is a need to gain a deeper understanding of workplace violence within the ambulance service so that future interventions may be based on current knowledge.

### Aim

To measure the 1 year cumulative incidence of and analyse the risk factors associated with workplace violence directed towards the ambulance service in a Swedish region.

## Methods

### Design

This was a prospective cohort study.

### Study setting

The Region of Örebro, Sweden, contains both urban and rural areas covering a total of 8500 km² with approximately 300 000 inhabitants, of which 125 000 live in the city of Örebro. There is a level-one trauma centre and two additional hospitals in the region. Depending on the time of day, there are between 12 and 17 ambulances available and 203 employees with a mean age of 43.9 years (male: 48.4 years, female: 38.5 years). Each ambulance is manned by two ambulance personnel of which one is stipulated to be a registered nurse,^28^ the second is not regulated, but is typically also a nurse but can in some cases be an emergency medical technician. In general, in the Region of Örebro, both of the ambulance personnel are typically nurses. At least one is a nurse specialised in prehospital emergency care, which encompasses a 3-year university course to become a registered nurse and an additional year of university studies for the specialisation. The emergency medical communication centre (EMCC) receives emergency calls and prioritises and dispatches ambulances.

### Study material

Data was collected from all ambulance missions within the region during the span of 1 year (5 May 2020 to 4 May 2021). The ambulance services used a digital medical record system (Ortivus Mobimed 4.69.1) to document all medical data such as patient data, vital signs, provided care and data on the ambulance assignment such as spatial data, response times and distances. Data was documented by the ambulance personnel using a tablet or computer. The digital medical record system had predetermined documentation fields and some of these were mandatory to fill in for each mission, while other were dependent on the mission and situation.

**Figure 1:**
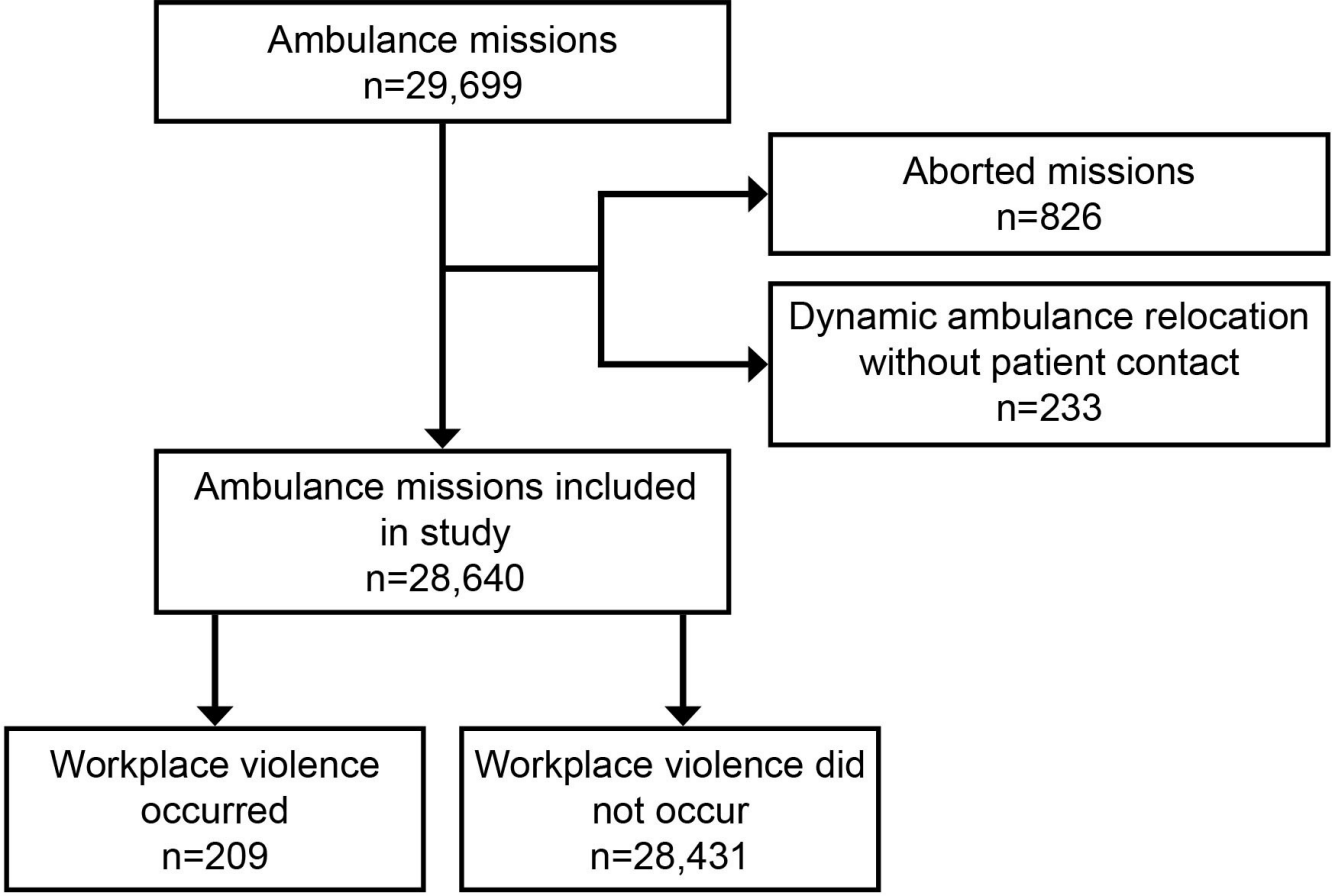
Flow chart of included and excluded ambulance missions.

Ambulance missions in which the ambulance personnel did not care for a patient (such as dynamic ambulance location requests or if no patient could be found at the scene) were excluded from the analysis (see figure 1).

A study-specific questionnaire was incorporated into the digital ambulance medical record system and completed by ambulance personnel for all ambulance missions during the study period. It was mandatory to answer the questionnaire.

### Data collection procedures

The digital study-specific questionnaire (see Supplementary Document 1) was developed by the research team who had both clinical and academic experience in prehospital emergency care in addition to experience in criminology. The occurrence of workplace violence was documented, as a mandatory question, for each ambulance mission during the study period. If workplace violence had occurred, the ambulance personnel were prompted to answer questions relating to the incident, its circumstances and the demographics for ambulance personnel exposed, see Supplementary Document 1.

The questionnaire was piloted among ambulance personnel before the study start, evaluating both content and technical solutions. No major changes were made after the pilot tests.

In addition to the data from the study-specific questionnaire, data on date, time for dispatching and medical reason for dispatching the ambulance were extracted from the digital ambulance medical record. The data was extracted as a Microsoft Excel spreadsheet (Microsoft Corporation, 2016) and imported into IBM SPSS (IBM Corporation, 2022).

### Analysis

Data was analysed using descriptive and analytic statistics. The incidence of workplace violence and its characteristics were described numerically and with percentages. Comparisons between groups were performed using Pearson’s x² test. Univariate and multivariate logistic regression was used to test for the association between independent variables and workplace violence. A p value <0.05 was considered statistically significant. Aborted missions and dynamic ambulance missions were not included in the analysis. Missing data was handled using complete-case analysis.^29^

### Patient and public involvement

There were no patients or public persons involved in the conceptualisation, data collection, analysis or writing and editing the manuscript.

### Ethical considerations

The study was approved by the Swedish Ethical Review Authority (document number 2019–04961). Research data was stored as part of the digital medical record system in which all study-specific information was marked as research data, meaning that it was not available to anyone other than the research team. The anonymised data set was stored on specific research data servers protected by two-factor authentication in accordance with standard routines.

## Results

There was a total of 29 699 ambulance missions during the study period, and 1059 were excluded (see figure 1), resulting in a total of 28 640 ambulance missions included in the study.

### One year cumulative incidence of workplace violence

Workplace violence occurred in 209 ambulance missions, resulting in a 1 year cumulative incidence of 0.7%. More than one type of violence occurred during the same ambulance mission in 30 of the incidences. The ambulance personnel were exposed to workplace violence, in some form, 239 times. Non-physical violence was most common (n=183, 76.6%) compared with physical violence (n=56, 23.4%).

Workplace violence was more common in the evening (14:00 to 22:00). One in five assignments was dispatched to a patient’s home. Workplace violence occurred more frequently in a private residence (n=110). However, the ambulance personnel were more likely to be exposed to workplace violence when caring for patients in a public place (1.4%) as compared with private residence (0.6%), p<0.001.

The most common reasons for dispatching an ambulance when workplace violence occurred were ‘intoxication or overdose’, ‘mental illness/risk for suicide’ or when the EMCC had difficulties defining the medical needs, an occurrence coded as ‘uncertain information’.

Workplace violence was more common when the ambulance personnel considered the patient to be suffering from mental illness or under the influence of alcohol or drugs (see table 1).

**Table 1:** Ambulance mission data

		All missionsn=28 640n (%)	Missions where WPV occurredn=209n (%)	Missions where no WPV occurredn=28 431n (%)	P value
Weekday	Monday to Thursday	16 116 (56.3)	108 (51.7)	16 008 (56.3)	0.18
	Friday to Sunday	12 524 (43.7)	101 (48.3)	12 423 (43.7)	0.18
Daytime	Daytime (06 to 14)	13 096 (45.7)	64 (30.6)	13 032 (45.8)	<0.01
	Evening (14 to 22)	10 746 (37.5)	104 (49.8)	10 642 (37.4)	<0.01
	Night time (22 to 06)	4798 (16.8)	41 (19.6)	4757 (16.7)	0.27
Dispatch location	Public place	5517 (22.4)	79 (41.8)	5438 (22.3)	<0.01
	Private residence	19 059 (77.5)	110 (58.2)	18 949 (77.8)	<0.01
	Missing*	4064	20	4044	
Patient gender	Male	13 893 (50.4)	113 (60.8)	13 780 (50.3)	0.11
	Female	13 688 (49.6)	73 (39.2)	13 615 (49.7)	
	Missing*	1059	23	1036	<0.01
Patient age	<18 years	1510 (5.5)	11 (5.9)	1499 (5.5)	1.0
	18–29 years	2438 (8.8)	44 (23.7)	2394 (8.7)	<0.01
	30–39 years	1972 (7.2)	28 (15.1)	1944 (7.1)	<0.01
	40–49 years	2043 (7.4)	18 (9.7)	2025 (7.4)	0.40
	50–59 years	2943 (10.7)	35 (18.8)	2908 (10.6)	<0.01
	60–65 years	1944 (7.1)	12 (6.5)	1937 (7.1)	0.55
	65 or more	14 718 (53.4)	38 (20.4)	14 680 (53.6)	<0.01
	Missing*	1072	23	1044	
Ambulance personnel’s assessment	Patient under the influence of alcohol or drugs	2210 (8.6)	131 (62.7)	2079 (7.3)	<0.01
	Missing*	3068	22	3046	
	Patient suffering from mental illness	1649 (6.5)	111 (53.1)	1538 (5.4)	<0.01
	Missing*	3150	27	3123	
	Communication difficulties between patient and ambulance personnel	1096 (4.3)	28 (13.4)	1068 (3.8)	<0.01
	Missing*	3155	29	3126	
EMCC dispatch category†	Intoxication or overdose	649 (2.3)	38 (18.1)	611 (2.1)	<0.01
	Mental illness/risk for suicide	707 (2.5)	32 (15.3)	675 (2.4)	<0.01
	Uncertain information	2441 (8.5)	30 (14.3)	2411 (8.5)	<0.01
	Unconsciousness (adult)	1216 (4.2)	21 (10.0)	1195 (4.2)	<0.01
	Violence/abuse	237 (0.8)	16 (7.7)	221 (0.8)	<0.01
	Minor trauma	3162 (11.0)	13 (6.2)	3149 (11.1)	0.03
	Seizures	630 (2.2)	8 (3.8)	622 (2.2)	0.11
	Breathing difficulties	3231 (11.3)	8 (3.8)	3223 (11.3)	<0.01
	Chest pain	3696 (12.9)	7 (3.4)	3689 (13.0)	<0.01
	Abdominal pain	2326 (8.1)	7 (3.4)	2319 (8.2	0.01

* Complete-case analysis. Missing data not included in analysis.

† EMCC assessed reason for dispatching an ambulance.

EMCCemergency medical communication centreWPV, workplace violence

### Type of workplace violence

Non-physical violence was more common compared with physical violence. Threatening gestures or body language were the most common forms of non-physical violence. Hitting was the most common form of physical violence (see table 2).

**Table 2:** Type of workplace violence and information on offender

Type of workplace violence*		N=209n(%)
Non-physical workplace violence†		183 (87.6)
	Threatening gestures or body language	77 (36.8)
	Verbal threats of violence	49 (23.4)
	Offensive comments	39 (18.7)
	Other	17 (8.1)
	Verbal death threats	12 (5.7)
	Offender persuades ambulance personnel	10 (4.8)
Physical workplace violence†		56 (26.8)
	Hit	18 (8.6)
	Held	11 (5.3)
	Material damages	11 (5.3)
	Other	41 (19.6)
Number of offenders		n=209n(%)
	One	170 (81.3)
	Two or more	14 (6.7)
	Missing	25 (12.0)
Offender profile		n=209n(%)
Gender		
	Male	133 (63.7)
	Female	55 (26.3)
	Animal	5 (2.4)
	Missing	16 (7.7)
Age		n=209n(%)
	<18 years	7 (3.3)
	18–29 years	42 (20.1)
	30–39 years	33 (15.8)
	40–49 years	16 (7.7)
	50–59 years	30 (14.4)
	60–65 years	10 (4.8)
	>65 years	30 (14.4)
	Missing	41 (19.6)
Offender		n=209n(%)
	Patient	164 (78.5)
	Relative	19 (9.1)
	Animal	5 (2.4)
	Bystander	4 (1.9)
	Other healthcare personnel	1 (0.5)
	Missing	16 (7.7)
Ambulance personnel perceived reason why WPV occurred‡		n=209n(%)
	The patient was under the influence of alcohol or drugs	131 (62.7)
	Mental illness	111 (53.1)
	Dissatisfied patient or bystander	42 (20.1)
	Aggressive due to somatic reasons	32 (15.3)
	Communication difficulties	28 (13.4)

* Ambulance personnel could be exposed to more than one type of workplace violence during the same mission.

† Unique events of physical – or non-physical workplace violence.

‡ Ambulance personnel could perceive more than one type of reason why workplace violence occurred.

WPVworkplace violence

### Ambulance personnel exposure

In all, 153 male and 114 female ambulance personnel reported workplace violence exposure. Male ambulance personnel between 30 and 39 years (n=56) and females between 18 and 29 years (n=36) were the most common victims of the workplace violence incidents. Three of the ambulance personnel were injured as a consequence of workplace violence, but none required treatment by a physician.

### Factors associated with the occurrence of workplace violence

Ambulance personnel reported patient dissatisfaction (n=42, 20%), most often related to different expectations of care (n=22, 11%), as the most common interpretation of why workplace violence occurred. In one mission, the long waiting time before the ambulance arrived on-scene was believed to be the reason why the workplace violence occurred. In 63.6% of all incidents (n=133), the offender was a male, and in 26.3% (n=55), the offender was a female.

The variables with the highest explanatory power for the outcome of workplace violence were patients considered to be suffering from mental illness or under the influence of alcohol or drugs: OR=6.099, p<0.001 and OR=6.096, p<0.001, respectively. Communication difficulties also demonstrated a significant difference in odds in relation to workplace violence (OR=2.139, p.003).

The dispatch category ‘violence/abuse’ displayed the highest association with the outcome (OR=4.388, p<0.001). Overall, the multivariable logistic regression showed a low explanatory power in predicting the occurrence of workplace violence (R²=0.268) (see table 3).

**Table 3:** OR for workplace violence, univariable and multivariable regression analyses

Independent variables	Univariable logistic regression			Multivariable logistic regression		
	Sig.	Exp(B)	95% CI	Sig.	Exp(B)	95% CI
Male (patient sex)	<0.01	1.53	1.14 to 2.06	0.11	1.33	0.94 to 1.88
Patient age						
<18 years	1.00	1.00	0.54 to 1.84	ref	ref	ref
18–29 years	<0.01	2.90	2.07 to 4.06	0.67	0.84	0.37 to 1.89
30–39 years	<0.01	2.11	1.41 to 3.15	0.53	0.76	0.32 to 1.79
40–49 years	0.41	1.23	0.76 to 2.00	0.090	0.45	0.18 to 1.13
50–59 years	<0.01	1.77	1.23 to 2.54	0.51	0.76	0.33 to 1.74
60–65 years	0.55	0.84	0.47 to 1.50	0.27	0.57	0.20 to 1.57
>65 years	<0.01	0.21	0.15 to 0.30	0.46	0.73	0.33 to 1.66
Ambulance personnel perceived reason why WPV occurred						
Patient under the influence of alcohol or drugs	<0.01	26.22	19.12 to 35.97	<0.01	6.10	3.85 to 9.66
Patient suffering from mental illness	<0.01	24.16	17.86 to 32.69	<0.01	6.10	3.95 to 9.42
Communication difficulties	<0.01	4.18	2.78 to 6.29	<0.01	2.14	1.31 to 3.50
Location of ambulance mission						
Public place	0.01	2.50	1.87 to 3.35	0.20	1.27	0.88 to 1.83
Time of day						
06.00–14.00	<0.01	0.52	0.39 to 0.70	ref	ref	ref
14.00–22.00	<0.01	1.66	1.26 to 2.17	0.51	1.14	0.78 to 1.67
22.00–06.00	0.27	1.22	0.86 to 1.71	0.57	1.15	0.71 to 1.85
EMCC dispatch category*						
Intoxication or overdose	<0.01	10.12	7.06 to 14.51	0.03	1.98	1.06 to 3.68
Unconsciousness (adult)	<0.01	2.55	1.62 to 4.01	0.07	1.92	0.95 to 3.87
Uncertain information/severely ill patient	<0.01	1.81	1.23 to 2.67	0.03	1.93	1.05 to 3.53
Suspicion of suicide/psychiatric patient	<0.01	7.43	5.06 to 10.92	0.07	1.83	0.96 to 3.47
Minor trauma/wounds/extremities	0.03	0.53	0.30 to 0.94	1.00	1.00	0.46 to 2.17
Violence/abuse	<0.01	10.58	6.25 to 17.92	<0.01	4.39	1.91 to 10.09
Breathing difficulties	<0.01	0.31	0.15 to 0.63	0.43	0.67	0.26 to 1.78
Chest pain	<0.01	0.23	0.11 to 0.49	0.12	0.42	0.15 to 1.23
Abdominal pain	0.02	0.39	0.18 to 0.83	0.64	1.24	0.50 to 3.07
Seizures	0.11	1.78	0.87 to 3.62	0.17	1.87	0.77 to 4.52

Logistic regression, Nagelkerke R²=0.268 for multivariable regression.

* EMCC assessed reason for dispatching an ambulance .

EMCCemergency medical communication centreWPVworkplace violence

## Discussion

The 1 year cumulative incidence of workplace violence within the ambulance services was 0.7%. Non-physical violence was more common than physical violence. Most offenders were men. There was a higher OR of workplace violence if the patient was under the influence of alcohol or drugs, suffering from mental illness or if there were communication difficulties. The dispatch categories ‘intoxication’, ‘uncertain information’ and ‘alarms regarding abuse’ were associated with the occurrence of workplace violence.

The 1 year cumulative incidence of workplace violence in the current study was low in comparison to prior studies.^13^,^15^ The explanation is probably multifactorial, such as in the difference in study design and catchment area. Other studies^12 14 15^ reporting on workplace violence incidence within the ambulance services have, however, had a retrospective design which may be influenced by recall bias.^30^ This was a prospective study in which data was recorded close to the actual ambulance missions using a mandatory questionnaire. This enabled rigorous data collection and allowed less risk of recall bias.^30^

The highest OR for workplace violence was observed for patients under the influence of alcohol or drugs or suffering from mental illness. This result is supported by previous research.^8 31 32^ However, it should be noted that many patients who were under the influence of alcohol or drugs or suffering from mental illness did not expose the ambulance personnel to workplace violence. When studying the risk factors for workplace violence, missions where no violence occurred should also be measured, reported and analysed.

Workplace violence occurs as a result of human, equipment, operational or social environmental factors.^33^ This describes a complexity that might explain why the variables in this study only provided a small part of the variation.^33^ Therefore, more research on factors indicating an increased risk for workplace violence is needed to develop advanced warning systems. Also, research on effective strategies to mitigate and reduce the occurrence of workplace violence is needed. However, the findings in this study can serve as an indication that ambulance personnel should maintain a mindset of safety when approaching a person under the influence of alcohol or drugs or suffering from mental illness.

### Method discussion/limitations

Strengths of the study were the prospective cohort design and integrating a questionnaire into the mandatory ambulance records reducing the risk of recall bias and missing data. A limitation was the lack of information about the age and sex of the ambulance personnel for missions where workplace violence did not occur. Neither, data on the individual exposure for workplace violence (eg, if some ambulance personnel were more exposed than others) was gathered. For future studies, this data should be recorded to enable comparisons.

Some variables (ie, mental illness or influence of alcohol or drugs) were reported based on clinical judgement by the ambulance personnel and not measured objectively, for example, by an alcometer. However, ambulance personnel are well-trained in making such clinical assessments.^34^

When performing comparisons between groups in the current study, there was a risk of multiple testing and inherently a risk of false positive results. As the cumulative incidence is small, we have chosen not to perform a correction for multiple testing but rather view the current study as hypothesis generating and needing replication.

Previous studies have identified certain geographic areas^35^ and the ambulance personnel’s behaviour^36^ and response time^37^ as additional risk factors. It would have been desirable to include these risk factors in the questionnaire as well, but, due to technical difficulties and lack of measurable data available, these variables could not be obtained. Also, since the data collection was performed during actual ambulance missions, it was important to reduce the time needed to answer all questions.

The current study was conducted during the COVID-19 pandemic, which could have affected the 1 year cumulative incidence. Conversely, many of the incidents of workplace violence in this study occurred in public areas. During the COVID-19 pandemic, restaurants, nightclubs and other public places were closed, which may have reduced the number of incidents for the ambulance service.

Since this study covered one of the 21 regions in Sweden, generalisation can be questioned. However, the studied region included both urban and rural areas and areas with a high occurrence of criminality and socioeconomic vulnerability. For future studies, using a similar study design in other geographical areas and during a non-pandemic setting would add value to determining the incidence and risk factors for workplace violence within the ambulance services.

### Conclusion

The 1 year cumulative incidence of workplace violence within the regional ambulance service was low in comparison to that of previous research. The overall regression model had low explanatory power, indicating that the phenomenon is complex and that additional variables need to be taken into account when trying to predict when workplace violence will occur. Additional research is needed to fully understand why workplace violence within the ambulance service occurs and how to mitigate such situations.

## supplementary material

10.1136/bmjopen-2023-074939 online supplemental file 1

## Data Availability

Data may be obtained from a third party and are not publicly available.
