# Supplementary material for: One year cumulative incidence and risk factors associated with workplace violence within the ambulance service in a Swedish region: a prospective cohort study
Source: BMJ Open. 2024 Sep 5;14(9):e074939. doi: 10.1136/bmjopen-2023-074939 (PMC11381691; doi:10.1136/bmjopen-2023-074939)
Supplement: online supplemental file 1 [file bmjopen-14-9-s001.pdf]

Supplementary file 1. Study-specific questionnaire incorporated in the medical records system

1. Did any of the following occur during the mission?*

a) Verbal threats or harassment? **YES/NO***

b) Physical violence or damage to/theft of any property? **YES/NO***

** If **no** workplace violence had occurred, the study person was directed to question no. 15.*
If workplace violence had occurred, the following questions were asked:

2. If YES on **a) verbal threats or harassment**: Choose the ones that best match what happened: verbal threats / harassment / sexual harassment / frightening behaviour

b) If the answer was **'yes'** to **'verbal threats'**: Please indicate whether the event including the following:

a) Verbal threats of violence YES/NO
b) Verbal death threats YES/NO
c) Threats of reporting the ambulance personnel YES/NO
d) Threats of notifying the media YES/NO

c) How was the event perceived? Not unpleasant / a little unpleasant / quite unpleasant / very unpleasant / extremely unpleasant or fear of death

d) If the answer was **'yes'** to '**harassment'**: Please indicate whether the event including the following:

a) Offensive comments YES/NO
b) Harassment due to ethnic background YES/NO
c) Harassment due to religious beliefs YES/NO

e) How was the event perceived? Not unpleasant / a little unpleasant / quite unpleasant / very unpleasant / extremely unpleasant or fear of death

f) If the answer was 'yes' to 'sexual harassment': Please indicate whether the event including the following:

a) Sexual innuendos through comments or gesturer YES/NO
b) Unwelcome sexual courtship YES/NO
c) Derogatory or offensive jokes with sexual undertones YES/NO
d) Caressing or pawing (excluding genitals, breasts or bottom) YES/NO

g) How was the event perceived? Not unpleasant / a little unpleasant / quite unpleasant / very unpleasant / extremely unpleasant or fear of death

h) If the answer was **'yes'** to '**to frightening behaviour'**: Please indicate whether the event including the following:

a) Threatening movements or body language YES/NO
b) Weapon threat YES/NO
c) Pursuit YES/NO

i) How was the event perceived? Not unpleasant / a little unpleasant / quite unpleasant / very unpleasant / extremely unpleasant or fear of death

3. If YES on **a) p*hysical violence or damage to / theft of any property***: Choose the ones that best match what happened: Physical abuse / damage to or theft of property / sexual assault / frightening behaviour

b). If the answer was **'yes'** to **'damage to / theft of property'**: Please indicate whether the event including the following:

a) Material damage YES/NO
b) Theft YES/NO

c) How was the event perceived? Not unpleasant / a little unpleasant / quite unpleasant / very unpleasant / extremely unpleasant or fear of death

d) If the answer was **'yes'** to '**sexual assault'**: : Please indicate whether the event including the following:

a) Unwanted touching of breasts, buttocks and/or genitals YES/NO
b) Unwanted kisses YES/NO
c) Forced sexual act YES/NO
d) Rape YES/NO

e) How was the event perceived? Not unpleasant / a little unpleasant / quite unpleasant / very unpleasant / extremely unpleasant or fear of death

4. How many offenders were there? (1/2/3/4 or more)

5. Who was/were the offender(s)?

a) The patient YES/NO
b) A relative YES/NO
c) A bystander YES/NO
d) Other healthcare personnel YES/NO
e) An animal YES/NO
f) Other YES/NO

6. The offender was…

a) Female YES/NO
b) Male YES/NO
c) Unclear YES/NO
d) An animal YES/NO

7. How old do you estimate the offender(s) to be? Less than 18 / 18–29 / 30–39 / 40–49 / 50–59 / 60–65 / more than 65

8. Who was exposed to the workplace violence?

a) Myself YES/NO
b) My colleague YES/NO
c) Other healthcare personnel YES/NO
d) The patient

9. The victim was….

a) Female YES/NO
b) Male YES/NO
c) Unclear YES/NO

10. How old was the victim? Less than 18 / 18–29 / 30–39/ 40–49 / 50–59 / 60–65 / more than 65

11. Where did the workplace violence occur?

a) In a private residence YES/NO
b) In a public place YES/NO

If YES: a) Public place inside
b) Public place, outside
c) Inside the ambulance
d) At the hospital
e) Other

12. Did the workplace violence lead to any consequences?

a) Interrupted examination or treatment YES/NO
b) Change to other means of transport to hospital (e.g. police transport) YES/NO
c) Denial of ambulance care YES/NO
d) Physical injuries to the ambulance personnel YES/NO

If YES: Did the injures require medical care? YES/NO

d) Fear or discomfort of the ambulance personnel YES/NO
e) Physical harm to the patient YES/NO

13. What do you think caused the occurrence of workplace violence?

a) The offender was under the influence of alcohol or drugs YES/NO
b) The offender was suffering from mental illness YES/NO
c) The patient was aggressive due to a somatic condition (e.g. hypoxia, pain or hypoglycaemia) YES/NO
d) The patient or a relative was dissatisfied with the care provided YES/NO (If YES: What was the dissatisfaction attributed to a) long waiting time, b) different expectation of care, c) triage, d) other)
e) Other YES/NO

14. Were there any communication difficulties? YES/NO

If YES: What kind of communication difficulties were there? a) language, b) hearing problems, c) neuropsychiatric problems, d) other

*Questions asked if **NO workplace violence** had occurred (NO on questions 1a and 1 b):*

15. According to your assessment, did you perceive any of the following conditions among the patient / circumstances during the mission?

a) Alcohol or drugs YES/NO

b) Mental illness YES/NO
c) An aggressive patient due to a somatic condition (e.g. hypoxia, pain or hypoglycaemia) YES/NO
d) Patients or relatives were dissatisfied with the care provided YES/NO

If YES: The dissatisfaction was attributed to: a) long waiting time, b) different expectation of care, c) triage, d) other)

16. Were there any communication difficulties? YES/NO

If YES: What kind of communication difficulties were there? a) language, b) hearing problems, c) neuropsychiatric problems, d) other

17. Were there any pets on site? (dog, cat, etc.) YES/NO

If YES: What kind of pet was it? A) dog, b) cat, c) other)

18. Where was the patient encountered? Private place / Public place

If a public place: a) inside, b) outside, c) hospital, c) health care centre, d) other
