# Supplementary material for: One year cumulative incidence and risk factors associated with workplace violence within the ambulance service in a Swedish region: a prospective cohort study
Source: BMJ Open. 2024 Sep 5;14(9):e074939. doi: 10.1136/bmjopen-2023-074939 (PMC11381691; doi:10.1136/bmjopen-2023-074939)
Supplement: Supplementary file 2 [file bmjopen-2023-074939.reviewer_comments.pdf]

PEER REVIEW HISTORY

BMJ Open publishes all reviews undertaken for accepted manuscripts. Reviewers are asked to complete a checklist review form and are provided with free text boxes to elaborate on their assessment. These free text comments are reproduced below.

ARTICLE DETAILS

**TITLE (PROVISIONAL)**	One year cumulative incidence and risk factors associated with workplace violence within the ambulance service in a Swedish region a prospective cohort study.
**AUTHORS**	Viking, Magnus; Hugelius, Karin; Höglund, Erik; Kurland, Lisa

VERSION 1 - REVIEW

**REVIEWER NAME**	Taylor, Jennifer
**REVIEWER AFFILIATION**	Drexel University School of Public Health, Department of Environmental & Occupational Health
**REVIEWER CONFLICT OF INTEREST**	I declare that I understand the above and have no competing interests.
**DATE REVIEW RETURNED**	21-Jul-2023

**GENERAL COMMENTS**	Abstract<br><br>Objective: “prevalence”. This should be “incidence” as the authors are measuring new missions where violence happened over all missions without WPV in one year.<br><br>Background<br><br>The background is incredibly brief and not at all comprehensive. For example, it lacks citations of several systematic reviews that have been conducted on violence against first responders. Furthermore, the background is rather dated and cites statistics that may not be relevant today. The introduction makes broad assessments based on one or two references. One study does not make a trend. Finally, the statements about ambulance personnel having the highest rates of occupational injury and fatality, and the fact that the author cites these incidents are increasing are not supported by the references cited. If this manuscript is to be revised it should have a much more robust, comprehensive, and contemporary literature review.<br><br>Study Material<br><br>The digital ambulance medical record system needs to be described in more detail.<br><br>Data collection procedures<br><br>Supplementary document 1: this flow chart of options depending on whether violence was experienced or not needs to be streamlined.

	Perhaps this can be recreated as a flow diagram. The repetition is not useful for the reviewer.<br><br>This section is missing a description of the data source for worker demographics and exposure (refer to the narrative at the bottom of page 8)<br><br>Analysis<br><br>This section was particularly brief and did not discuss that they conducted a univariate and multivariable logistic regression specifically.<br><br>The mention of the STROBE guidelines for cohort studies on line 31, is not analysis and should not be included in the methods. The STROBE checklist is to be conducted by the authors after their manuscript is complete to show that they have clearly and transparently written up their research according to accepted guidelines for scientific clarity.<br><br>Results<br><br>Line 36 to 37 (describing where workplace violence was most frequent): The authors say it was most frequent in public places, but after reviewing table one this is not the case. In actuality, private residences were more commonly the place of workplace violence (52.6%) vs 37.8% public. Indeed overall the location was overwhelmingly in a private residence versus a public place. This also is demonstrated in the regressions of table 3 where a public place is actually protective in the odds ratio. This needs to be corrected throughout the manuscript from the abstract through the discussion.<br><br>Because the authors collected verbal and physical violence and cases of both, their descriptive statistics on line 35 should include all of those. Currently only verbal and physical are described.<br><br>Table 1: the EMCC dispatch category results should be put in descending order so that it is very easily seen what the most common reasons for dispatch were. It would make sense to use the column with workplace violence missions to be the primary sorting column because it is the key outcome in the study.<br><br>Tables 1 and 2: the titles should include the data source. It is unclear what came from the digital ambulance medical record system versus the supplemental survey.<br><br>On page 8 at the bottom with the heading “ambulance personnel exposed” this reviewer questions where did this data come from. It is not in any of the tables and is not on the supplemental form.<br><br>The last sentence says that “three of the ambulance personnel were injured as a consequence of workplace violence but none require treatment by a physician (see table 2)”. However, none of this data appears in table 2. This becomes even more confusing because on page 11 in the discussion of limitation’s (first paragraph), the authors describe a limitation in the lack of data about the ambulance personnel ,but on page 8 they are citing data. At this point I began to

lose faith that the authors really had a good handle on what they had done and that I could have confidence in the results. Overall there are some rather careless and confusing moments in the presentation of the results and in the writing of them.

On page 9 at the end of the results the authors describe the low explanatory power of the R squared but do not give a citation to express the standard by which this would be. It was also not stated that this was the unadjusted or adjusted R squared. (This reviewer had to look at the reference at the bottom of Table 3 to find this out). The authors should show an adjusted and unadjusted R squared. If the difference between them is not □.05 per Nagelkerke - plus other criteria proposed by Riley et al (https://www.ncbi.nlm.nih.gov/pmc/articles/PMC6519266/), - then the model may be overfitted. This could be the case given that there are five variables for only 209 events and by accepted practice they would need around 500 observations in order to avoid collinearity issues.

Table 3: please see that “public place” had a protective odds ratio compared to private place and adjust the discussion and the manuscript appropriately.

Given the low incident rate determined from the study, did the authors consider human factors such as “drop down” awareness and fatigue with the supplemental survey? This is not described in the manuscript, nor is any testing that they did to be sure that participants would go through all of the survey as appropriate. It is known in this industry that respondents will work around drop downs in order to avoid spending more time on data entry.

On page 11 line 28 in the subheader of the discussion on “method discussion\limitations” the second sentence describes the limitation of not having ambulance personnel demographic data but it is then described earlier in the results section on page 8.

Around line 46 in the same section the fourth paragraph, second sentence the authors discuss that workplace violence occurred in public areas more often than private. This is not the case given the data that they presented and needs to be corrected throughout the manuscript. Their conclusions need to be adjusted as well.

References

There are numerous references that are incomplete. For example references 1 and 13. This reviewer did not go through all of them, but they should all be checked for completeness.

This reviewer found interesting and important results from the study:

Table 1: that the investigators included communication difficulties in the ambulance personnel’s assessment was very useful information and not commonly assessed in workplace violence studies. The discussion of patient satisfaction was also very important and useful. This is a strength of the study.

That intoxication and mental health led the list of reasons for dispatch also makes sense given this reviewers knowledge of other studies.

**REVIEWER NAME**	Helmer, Jennie
**REVIEWER AFFILIATION**	The University of British Columbia
**REVIEWER CONFLICT OF INTEREST**	None
**DATE REVIEW RETURNED**	02-Aug-2023

**GENERAL COMMENTS**	Excellent insight into factors of workplace violence in the Ambulance Service in Sweden. While your findings may not be generalizable due to the study period and the unique model of EMS in Sweden, the methodology appears sound and could be replicated in other paramedic agencies.

VERSION 1 – AUTHOR RESPONSE

Reviewer comment	Authors´reply
**Reviewer #1:**	Thank you for the effort and time to review our article on workplace violence in Sweden. We have found that many of your comments are valuable and very important and have revised accordingly. We hope the changes and comments are of satisfaction.
**Abstract:**<br><br>Objective: "prevalence". This should be "incidence" as the authors are measuring new missions where violence happened over all missions without WPV in one year.	Thank you for this comment. We have consulted our epidemiologist and there appear to be different ways to see this. We have measured the one year cumulative incidence in relation to all ambulance missions.<br><br>Accordingly we have changed prevalence to one year cumulative incidence where suitable, as marked in the manuscript.
**Background:**<br><br>The background is incredibly brief and not at all comprehensive. For example, it lacks citations of several systematic reviews that have been conducted on violence against first responders.	Thank you for bringing this to our attention. Some of the data in the background are from systematic reviews that has been found to be incorrect and therefore references to original articles were used instead. We have added references and revised the background.

	The first emergency responders are predominantly ambulances in Sweden, which we are understand differs from that e.g. in the United States. This is the motivation for not including other professions in the background.
Furthermore, the background is rather dated and cites statistics that may not be relevant today.	Thank you for pointing this out. We agree; the data on WPV in the ambulance service is dated, especially in Sweden. In accordance with your suggestion above the background has been revised.
The introduction makes broad assessments based on one or two references. One study does not make a trend.	Thank you for this important comment. We have revised accordingly.
Finally, the statements about ambulance personnel having the highest rates of occupational injury and fatality, and the fact that the author cites these incidents are increasing are not supported by the references cited. If this manuscript is to be revised it should have a much more robust, comprehensive, and contemporary literature review.	Thank you for this comment, which is in line with prior comments on the need of a revision of the background.<br><br>The background has been revised and additional 16 publications have been added to support the statements. Hopefully, these changes will make the background more comprehensive and clear.
**Study Material:**<br><br>The digital ambulance medical record system needs to be described in more detail.	Thank for this comment. The description was brief due to the word limit. However, due to the revision freeing up more words, we have included a more detailed description of the ambulance medical record system.
Data collection procedures:<br><br>Supplementary document 1: this flow chart of options depending on whether violence was experienced or not needs to be streamlined. Perhaps this can be recreated as a flow diagram. The repetition is not useful for the reviewer.	Thank you for this great suggestion. This has not been easy since there are many questions in the questionnaire. We thought about making a flow chart as suggested, but opted to make the questionnaire more streamlined as a whole. It has been shortened significantly with the aim to be easier to read. To

	be transparent, the supplementary material shows the complete questionnaire.
This section is missing a description of the data source for worker demographics and exposure (refer to the narrative at the bottom of page 8)	Thank for this comment. The data collection procedures-section has been revised.
**Analysis:**<br><br>This section was particularly brief and did not discuss that they conducted a univariate and multivariable logistic regression specifically.	Thank for the comment. We have revised this section and have now specified that both univariate and multivariable logistic regression were used.
The mention of the STROBE guidelines for cohort studies on line 31, is not analysis and should not be included in the methods. The STROBE checklist is to be conducted by the authors after their manuscript is complete to show that they have clearly and transparently written up their research according to accepted guidelines for scientific clarity.	Thank you for clarifying this. We have deleted this section.
**Results:**<br><br>Line 36 to 37 (describing where workplace violence was most frequent): The authors say it was most frequent in public places, but after reviewing table one this is not the case. In actuality, private residences were more commonly the place of workplace violence (52.6%) vs 37.8% public. Indeed overall the location was overwhelmingly in a private residence versus a public place. This also is demonstrated in the regressions of table 3 where a public place is actually protective in the odds ratio. This needs to be corrected throughout the manuscript from the abstract through the discussion.	Thank you for notifying us on this matter. We have re-calculated and checked all numbers and revised (corrected) the table accordingly.<br><br>One variable needed to be corrected since it showed the inverse relationship. It is now clearer that "public place" contain more incidences concerning WPV.
Because the authors collected verbal and physical violence and cases of both, their descriptive statistics on line 35 should include all of those. Currently only verbal and physical are described.	If we understand the comment correctly, there is a lack in the description of how many events occurred when non-physical WPV and physical WPV occurred at the same time. This has been clarified and added. There were 30 events where physical and non-physical violence occurred in the same mission.
Table 1: the EMCC dispatch category results should be put in descending order so that it is very easily seen what the most common reasons for dispatch were. It would make sense to use the column with workplace violence missions to be the	Thank for the excellent suggestion. The section has been revised accordingly.

primary sorting column because it is the key outcome in the study.	
Tables 1 and 2: the titles should include the data source. It is unclear what came from the digital ambulance medical record system versus the supplemental survey.	Thank you for this notification. To clarify from where the data was extracted the method section has been revised to clarify this.
On page 8 at the bottom with the heading “ambulance personnel exposed” this reviewer questions where did this data come from. It is not in any of the tables and is not on the supplemental form.	Thank you for making us aware if this matter. The data is collected using the questionnaire under the question “did the workplace violence lead to any consequences?”. There are follow-up questions about “Physical injuries to the ambulance personnel” and whether the injuries required medical care. Also the demographics were collected from the questionnaire under “The victim was…” and “How old was the victim. We have chosen not to specify these numbers in the tables, since we wanted the tables to be as brief and understandable as possible. This is why we wrote the results in the text.
The last sentence says that “three of the ambulance personnel were injured as a consequence of workplace violence but none require treatment by a physician (see table 2)”. However, none of this data appears in table 2. This becomes even more confusing because on page 11 in the discussion of limitation’s (first paragraph), the authors describe a limitation in the lack of data about the ambulance personnel ,but on page 8 they are citing data. At this point I began to lose faith that the authors really had a good handle on what they had done and that I could have confidence in the results. Overall there are some rather careless and confusing moments in the presentation of the results and in the writing of them.	Thank you for noticing this. We agree that these mistakes in presenting data are serious, and we have carefully revised the manuscript. As mentioned in the comment above, we had to choose what data would fit and be suitable to present in the tables. We have removed the data about ambulance personnel and written it in the text instead. The “(see table 2)” has been removed. The lack of data does not refer to this section of demographics. It refers to the lack of data when WPV did not occur. Therefore, no comparisons of ambulance personnel could be done

	between cases where WPV did and did not occur.
On page 9 at the end of the results the authors describe the low explanatory power of the R squared but do not give a citation to express the standard by which this would be. It was also not stated that this was the unadjusted or adjusted R squared. (This reviewer had to look at the reference at the bottom of Table 3 to find this out). The authors should show an adjusted and unadjusted R squared. If the difference between them is not ≤.05 per Nagelkerke - plus other criteria proposed by Riley et al (https://www.ncbi.nlm.nih.gov/pmc/articles/PMC6519266/), - then the model may be overfitted. This could be the case given that there are five variables for only 209 events and by accepted practice they would need around 500 observations in order to avoid collinearity issues.	Thank you for bringing this up to discussion. We have consulted our statistician about this matter and we can't see any signs of overfitting in the results. The results are reasonable and interpretable. This article is made up of a standard use of logistic regression made in SPSS. To our knowledge this model fit measure is not possible to perform in SPSS.
Table 3: please see that "public place" had a protective odds ratio compared to private place and adjust the discussion and the manuscript appropriately.	Thank you for noticing this. This has been changed as described in previous comment.
Given the low incident rate determined from the study, did the authors consider human factors such as "drop down" awareness and fatigue with the supplemental survey? This is not described in the manuscript, nor is any testing that they did to be sure that participants would go through all of the survey as appropriate. It is known in this industry that respondents will work around drop downs in order to avoid spending more time on data entry.	Thank you for this comment. We agree that the questionnaire design is essential when conducting studies like this. No "drop down" alternatives were used.<br><br>The questionnaire was piloted before the study, and found easy to understand, complete and was not deemed time consuming.
On page 11 line 28 in the subheader of the discussion on "method discussion\limitations" the second sentence describes the limitation of not having ambulance personnel demographic data but it is then described earlier in the results section on page 8.	Thank for this comment. It refers to the limitation that we did not record, for example, if one ambulance personnel had been exposed to WPV several times, or the demographics of each ambulance personnel reporting being involved in WPV. This data was only available on group level (which is described in the manuscript). This is a significant limitation of the study, motivating why we think it needs to be highlighted and considered in future studies.

Around line 46 in the same section the fourth paragraph, second sentence the authors discuss that workplace violence occurred in public areas more often than private. This is not the case given the data that they presented and needs to be corrected throughout the manuscript. Their conclusions need to be adjusted as well.	Thank for noticing this. This has been corrected as commented above.
**References:**<br>There are numerous references that are incomplete. For example references 1 and 13. This reviewer did not go through all of them, but they should all be checked for completeness.	Thank you for noticing this. The references has been changed accordingly.
This reviewer found interesting and important results from the study:<br><br>Table 1: that the investigators included communication difficulties in the ambulance personnel's assessment was very useful information and not commonly assessed in workplace violence studies. The discussion of patient satisfaction was also very important and useful. This is a strength of the study.	We agree that communication difficulties are very interesting when trying to understand the cause of WPV and should be included in future research in order to be able to make interventions and mitigate WPV.<br><br>No change in manuscript.
That intoxication and mental health led the list of reasons for dispatch also makes sense given this reviewers knowledge of other studies.	We agree with the reviewer.<br><br>No change in the manuscript.
**Reviewer #2**	
Excellent insight into factors of workplace violence in the Ambulance Service in Sweden. While your findings may not be generalizable due to the study period and the unique model of EMS in Sweden, the methodology appears sound and could be replicated in other paramedic agencies.	Thank for your time and effort reading and commenting on our manuscript. We agree that the generalization is a limitation, which is also commented on in the manuscript. We also agree that the methodology could be helpful to when designing future studies.
**Additional changes to the manuscript**	Under the Mehods section the authors have removed the part: "Due to a large number of missing values in some of the collected data (e.g., data covering how the ambulance personnel had experienced the workplace violence situation), these variables were deemed to be inadequate for statistical analysis.".

VERSION 2 - REVIEW

**REVIEWER NAME**	Helmer, Jennie
**REVIEWER AFFILIATION**	The University of British Columbia
**REVIEWER CONFLICT OF INTEREST**	N/A
**DATE REVIEW RETURNED**	03-Oct-2023

**GENERAL COMMENTS**	-Third paragraph, page 1 the sentence "Non-physical violence...". The sentence appears incomplete and requires revising.<br>-Study setting. Why is only the nurse role elucidated on? Assuming that their partner also has common educational requirements?<br>-No comments on statistical interpretations. Given other reviewer comment, an expert review of statistics, is likely appropriate.<br>-References appear complete

**REVIEWER NAME**	Fort, Emmanuel
**REVIEWER AFFILIATION**	University of Lyon, University of Lyon 1, University of Gustave Eiffel, UMRESTTE, UMR T_9405, F- 69373, LYON, France
**REVIEWER CONFLICT OF INTEREST**	None
**DATE REVIEW RETURNED**	04-Jan-2024

**GENERAL COMMENTS**	It is a really interesting pape about the workplace violence in the ambulance service.<br>The draft is well written, it was very pleaant to read it.<br>There are some weaknesses and some mistakes that need to be checked and corrected before a publication.<br>Analysis part (p31 line 45): It is a prospective cohort study. And in case of an incidence/prevalence study, when the outome is very low (0.7%), OR can not be a good estimation of the prevalence risk / relative risk. In this way, the authors should have performed a log-binomial regression to calculate crude or adjusted prevalence ratios (Crude PR / Adjusted PR), and their 95% confidence intervals for the evaluate the associated variables and the exposure workplace violence.<br>Results part (p32 line 26): 14:00 and 22:00 Please replace "and" by "to".<br>Table 1 : Why autors give the p--value for each modalities of each varaibles ? Give the global p-value of the Pearson’s chi-squared test for each comparisaon between variable and the dichotomous variable Exposure to workplace violence (the outcome in your paper).<br>P33 line 4: It seems that the title of the table2 is nnot add in the right place in the draft. It shoulb be move p34 line 2.<br>Ambulance personnel exposure part (p33 line 54) please delete the word "than" written between 114 and female<br>Table 2 (the table p34) Please add percentage in bracket after the frequency<br>Table 3 (p35) and Factors associated with the occurrence of workplace violence part (p35) : please corretc this part with using prevalence ratio instead of odds ratio. Moreover, no need to keep so digit in the numbers given in the table. 2 digit for the pravalence ratio and the 95% CI is enough.Please add the global p-value for each association between variables and outcome (Type III tests) and dont give the

VERSION 2 – AUTHOR RESPONSE

Reviewer comment	Authors´reply
**Reviewer #3:**<br><br>It is a really interesting paper about the workplace violence in the ambulance service. The draft is well written, it was very pleasant to read it.<br>There are some weaknesses and some mistakes that need to be checked and corrected before a publication.	Thank you for the kind words, and thank you for taking the time to read and comment on our paper. We have considered all suggestions and reply below, point by point.
Analysis part (p31 line 45): It is a prospective cohort study. And in case of an incidence/prevalence study, when the outcome is very low (0.7%), OR can not be a good estimation of the prevalence risk / relative risk. In this way, the authors should have performed a log-binomial regression to calculate crude or adjusted prevalence<br>ratios (Crude PR / Adjusted PR), and their 95% confidence intervals for the evaluate the associated variables and the exposure workplace violence	Thank you for commenting on this matter. We have collaborated with a statistician from the start, who has also taken part of the raw data. The analysis was planned with the statistician, and he has overseen each step. We considered other methods but chose the current method since it is common, robust, and interprets well. Furthermore, the data meets the requirements stipulated by the model.<br><br>No change in manuscript.
**Results:**	
Results part (p32 line 26): 14:00 and 22:00 Please replace "and" by "to".	Thank you for noticing this. It has been changed accordingly.
Table 1 : Why autors give the p--value for each modalities of each varaibles ? Give the global p-value of the Pearson’s chi-squared test for each comparisaon between variable and the dichotomous variable Exposure to workplace violence (the outcome in your paper).	We have considered your suggestion of a global p-value. We assume you mean a global p-value for each modality as a global p-value for e.g. age or gender.<br><br>However, we wanted to perform comparisons between the group exposed to workplace violence and those that had not been exposed on a more detailed level; e.g. comparisons between specific age categories and sex, male female. A global p-value would not suffice in this case.<br><br>The risk of inflated type I errors was mitigated through conservative interpretation, and no far-reaching conclusions were drawn from isolated significances.

	No change to manuscript.
P33 line 4: It seems that the title of the table2 is nnot add in the right place in the draft. It shoulb be move p34 line 2.	Thank you for pointing this out. We have revised the manuscript and made sure that the title of Table 2 is on top of the Table.
Ambulance personnel exposure part (p33 line 54) please delete the word "than" written between 114 and female	Thank you for the comment. The word "than" has been deleted per your suggestion.
Table 2 (the table p34) Please add percentage in bracket after the frequency	Thank you for pointing this out.<br><br>We have revised the manuscript so that percent is presented after the brackets, i.e. n(%).<br><br>We have also clarified (using *) that for some modalities, there are more than one option. This is exemplified by e.g., type of workplace violence: 209 is the total number of ambulance missions with workplace violence, but for each individual mission more that one type of workplace violence occurred. 209 is used as the denominator, leading to the sum of type of workplace violence being greater than 100%.
Table 3 (p35) and Factors associated with the occurrence of workplace violence part (p35) : please corretc this part with using prevalence ratio instead of odds ratio.	Thank you for this comment.<br>As discussed above, to present prevalence ration instead of OR is an alternative.<br><br>Based on your comments we considered the advantages and disadvantages with both types of analyses and discussed this again with our statistician. However, to be consistent with the above line of reasoning in response to your previous comment and statistical analysis, we have made not change in the manuscript and hope that this is acceptable.
Moreover, no need to keep so digit in the numbers given in the table. 2 digit for the pravalence ratio and the 95% CI is enough.	Thank you for this suggestion. We have approximated to two digits overall.
Please add the global p-value for each association between variables and outcome (Type III tests) and dont give the	Thank you for the comment. Unfortunately, the sentence is incomplete.<br><br>We do, however, suspect that you are, in line with your suggestion above, suggesting adding a global p-value but what else is unclear.

	We naturally agree that the risk for false positive associations increases with the number of comparisons.<br><br>Also here, we have consulted our statistician.<br><br>Adding a global p value would not add additional value to the reader in this case, but rather increase the inherent risk of multiple comparisons. And, as commented above, the risk of inflated type I errors was mitigated through conservative interpretation, and no far-reaching conclusions were drawn from isolated significances.<br><br>No change to manuscript.
**References:**	
**Reviewer #2**	
	Thank you for taking the time to revise our manuscript and for meaningful feedback.
Third paragraph, page 1 the sentence "Non-physical violence...". The sentence appears incomplete and requires revising.	Thank you for the comment. The sentence has been revised.<br><br>Change to manuscript: The sentence now reads:<br><br>*“Non-physical violence includes, e.g., verbal abuse, threats, sexual harassment and bullying (23, 24) and has been shown to be more common than physical violence (7, 9, 17, 25), motivating recording both physical and non-physical violence.”*
Study setting. Why is only the nurse role elucidated on? Assuming that their partner also has common educational requirements?	Thank you for the comment.<br><br>In accordance with a law passed in Sweden in 2005, it is mandatory that at least one of the ambulance personnel is a licensed health care provider. This has been solved by implementing

	that a registered nurse was mandatory as one of the two ambulance personnel.<br><br>Today most regions require that at least one of the two be a nurse specialized in prehospital care. The second professional is not regulated specifically. But it is most common that this person is a registered nurse, although some emergency medical technicians remain.<br><br>Revised manuscript now reads:<br><br>*“Each ambulance is manned by two ambulance personnel of which one is stipulated to be a registered nurse (28), the second is not regulated, but is typically also a nurse but can in some cases be an emergency medical technician. In general, in the Region of Örebro, both of the ambulance personnel are typically nurses. At least one is a nurse specialized in prehospital emergency care, which encompasses a three-year university course to become a registered nurse and an additional year of university studies for the specialization.”*
No comments on statistical interpretations. Given other reviewer comment, an expert review of statistics, is likely appropriate.	Yes, the manuscript has been reviewed by a third reviewer and several statistical comments have been responded to, see above.
References appear complete	-

VERSION 3 - REVIEW

**REVIEWER NAME**	Fort, Emmanuel
**REVIEWER AFFILIATION**	University of Lyon, University of Lyon 1, University of Gustave Eiffel, UMRESTTE, UMR T_9405, F- 69373, LYON, France
**REVIEWER CONFLICT OF INTEREST**	None
**DATE REVIEW RETURNED**	11-Mar-2024

**GENERAL COMMENTS**	Analysis part<br>I definitely disagree with the way your performed the statistical analysis. First it doesn't fit with the type of study. Secondly, it doesn't

	fot with the title of your draft where "risk factors" is clearly mentioned.<br><br>You can read the following references:<br>Robbins AS, Chao SY, Fonseca VP. What's the relative risk? A method to directly estimate risk ratios in cohort studies of common outcomes. Ann Epidemiol. 2002 Oct;12(7):452-4. doi: 10.1016/s1047-2797(01)00278-2. PMID: 12377421.<br><br>Sistrom CL, Garvan CW. Proportions, odds, and risk. Radiology. 2004 Jan;230(1):12-9. doi: 10.1148/radiol.2301031028. PMID: 14695382.<br><br>Diaz-Quijano FA. A simple method for estimating relative risk using logistic regression. BMC Med Res Methodol. 2012 Feb 15;12:14. doi: 10.1186/1471-2288-12-14. PMID: 22335836; PMCID: PMC3305608.<br><br>In the event of convergence problems that can occur in multivariable models, some approaches exist (Deddens, 2008).<br>Deddens JA, Petersen MR. Approaches for estimating prevalence ratios. Occup Environ Med. 2008 Jul;65(7):481, 501-6. doi: 10.1136/oem.2007.034777. PMID: 18562687.<br><br>Table 1 and method: Authors performed Pearson's chi-squared tests for comparisons between groups. These tests homogeneity compare the distribution of counts for two or more groups using the same categorical variable. In this way it is a global test for the variable testing.<br><br>Performing comparisons between the group exposed to workplace violence and those that had not been exposed on a more detailed level imply to perform as many tests as there are modalities in the variable. Doing that, authors did not take into consideration the multiplicity of tests with a correction for exemple.<br>Giving the global p-value for the comparison is enough.<br><br>Table 3: There is an issue with the presentation of the table 2 and the table 3. The top of the table 3 is overwritten on the end of the table 2. Moreiover, the end of the table 3 is on the next page. Please correct.<br><br>Table 3: I am confused but I did not see the reference modality for the following variables in the univariate step: Patient age and Time of day.<br><br>Same comment than for the table1. Please give to the reader the global p-value for the association between each variables and the outcome.

VERSION 3 – AUTHOR RESPONSE

**Reviewer comment**	**Authors´reply**
**Reviewer #3:**	

**Analysis**	
I definitely disagree with the way your performed the statistical analysis. First it doesn't fit with the type of study. Secondly, it doesn't fot with the title of your draft where "risk factors" is clearly mentioned.<br><br>You can read the following references:<br><br>Robbins AS, Chao SY, Fonseca VP. What's the relative risk? A method to directly estimate risk ratios in cohort studies of common outcomes. Ann Epidemiol. 2002 Oct;12(7):452-4. doi: 10.1016/s1047-2797(01)00278-2. PMID: 12377421.<br><br>Sistrom CL, Garvan CW. Proportions, odds, and risk. Radiology. 2004 Jan;230(1):12-9. doi: 10.1148/radiol.2301031028. PMID: 14695382.<br><br>Diaz-Quijano FA. A simple method for estimating relative risk using logistic regression. BMC Med Res Methodol. 2012 Feb 15;12:14. doi: 10.1186/1471-2288-12-14. PMID: 22335836; PMCID: PMC3305608.	We have taken your comment very seriously and discussed at length with our statistician.<br><br>We do understand that you are not pleased with the methodology. However, it is unclear specifically what and how you want us to change it.<br><br>Our statistician has taken part of the raw data, planned the analyses, and overseen each step from the beginning and is also an experienced statistician.<br><br>Despite our general attitude to revise according to reviewers' responses, we have chosen to maintain the methodology, ie applying logistic regression to address the study of the association of factors with the outcome of violence towards ambulance personnel and express this as odds ratio (uni- and multivariable) since it is a common and robust method and the results are understandable, in addition to the data meeting the requirements stipulated by the model, which is also supported by the reference Deddens et al.<br><br>We have taken part of the references you have suggested and find support in the reference Deddens et al. for the chosen method, i.e., a standard method using logistic regression which normally is calculated using binary logistic regression with OR. Diddens et al. points out that OR should be used for rare outcomes i.e. below 10%, which further supports the use of OR in the current study.<br><br>Furthermore, according to Deddens et al.: "Logistic regression yields maximum likelihood estimates (MLEs) of the OR (adjusted for other covariates). If the adjusted OR is the parameter of interest, then these MLEs are generally considered the best<br><br>estimators available."

	Reference: Deddens JA, Petersen MR. Approaches for estimating prevalence ratios. Occup Environ Med. 2008 Jul;65(7):481, 501-6. doi: 10.1136/oem.2007.034777. PMID: 18562687.<br><br>Thank you for pointing out that "risk factors" in the title can be misleading. We have therefore revised the title.<br><br>Revised manuscript title:<br><br>One year cumulative incidence and risk factors associated with workplace violence within the ambulance service in a Swedish region: a prospective cohort study.
In the event of convergence problems that can occur in multivariable models, some approaches exist<br><br>(Deddens, 2008).<br>Deddens JA, Petersen MR. Approaches for estimating prevalence ratios. Occup Environ Med. 2008 Jul;65(7):481, 501-6. doi: 10.1136/oem.2007.034777. PMID: 18562687.	Thank you for this comment.<br><br>However, there were no convergence problems using the current method.<br><br>No changes to manuscript.
**Table 1 and method:**	
Authors performed Pearson's chi-squared tests for comparisons between groups. These tests homogeneity compare the distribution of counts for two or more groups using the same categorical variable. In this way it is a global test for the variable testing.	Thank you for this comment. However, a global p-value calculation is not part of the Pearson's chi-square test.<br><br>No changes to manuscript.
Performing comparisons between the group exposed to workplace violence and those that had not been exposed on a more detailed level imply to perform as many tests as there are modalities in the variable. Doing that, authors did not take into consideration the multiplicity of tests with a correction Wfor exemple.<br>Giving the global p-value for the comparison is enough.	Thank you for this important comment. Yes, there is a risk of multiple testing which cannot be denied and inherently a risk of false positive results.<br><br>This is now added to and commented in the limitations section.<br><br>As the cumulative incidence is small we have chosen not to perform a correction for multiple testing but rather view the current study as

	hypothesis generating, and needs replication. This has been added to the limitations section.<br><br>Revised manuscript now reads:<br><br>"When performing comparisons between groups in the current study, there was a risk of multiple testing and inherently a risk of false positive results. As the cumulative incidence is small, we have chosen not to perform a correction for multiple testing but rather view the current study as hypothesis generating and needing replication. "
**Table 3:**	
There is an issue with the presentation of the table 2 and the table 3. The top of the table 3 is overwritten on the end of the table 2. Moreiover, the end of the table 3 is on the next page. Please correct.	Thank you for this comment, and our apologizes for the layout that appears when submitting the tables to the journal. However, our understanding is that these layout problems will be solved in the next step of the process. Otherwise, we kindly ask you to address this issue to the editor to enable a good review process should it be repeated.
I am confused but I did not see the reference modality for the following variables in the univariate step: Patient age and Time of day.	Thank you for the comment.<br><br>There is no reference modality in the univariable logistic regression since they are calculated one by one. There is a reference modality for: gender, patient age and time of day for the multivariable logistic regression in Table 3.
Same comment than for the table1. Please give to the reader the global p-value for the association between each variables and the outcome.	Thank you for the comment. However, we are not sure how to respond to this comment, as we are not sure what you are asking us to do.<br><br>Above, there are two comments (and answers) relating to Table 1. In the first you have asked for a global p-value, however, the results in Table 1 are calculated using Pearsons chi-square and not as those in Table 3 using logistic regression. To compute and present a global

	test for the multiple logistic regression would not answer the study aim.<br><br>The second comment in relation to Table 1 concerns multiple testing which has been answered above. In addition, adjusting for multiple comparisons is not standard practice for a multiple regression model.

VERSION 4 - REVIEW

**REVIEWER NAME**	Fort, Emmanuel
**REVIEWER AFFILIATION**	University of Lyon, University of Lyon 1, University of Gustave Eiffel, UMRESTTE, UMR T_9405, F- 69373, LYON, France
**REVIEWER CONFLICT OF INTEREST**	None
**DATE REVIEW RETURNED**	26-Jul-2024

**GENERAL COMMENTS**	Regarding the main question of methodology, I wanted to say that the best approach for a one-year follow-up is to perform relative risks using log-binomial regression. Of course, in some ways, calculating odd ratios with logistic regression is a good estimate of relative risk, but where technically possible, I prefer the use of relative risk because it fits better the data.<br>This kind of use of logistic regression in order to use log binomial regression is quite common is epidemiology, because in previous decades, log binomial regression models were difficult to use in multivariate analysis due to the limited power of computers. One way of doing this was often to use logistic regression or Cox regression by symmetry,odds ratio and hazard ratios could been good estimation of relative risks.<br>In this way, I think that the term "risk factors" in your manuscrit should be replaced by "determinants".<br><br>Table 1: Of course, a global p-value calculation for each variable can be performed with the Pearson's chi-square test. For exemple, the p-value for the association between Patient gender and the variable of interets in the table 1 is equal to 0.0045 (with a value of the chi-squared test equal to 8.0727).<br><br>Table 3 : I am confused by your answer about the lack of the reference modality for the following variables in the univariate step: Patient age and Time of day. Why did you answer that they were calculated one by one ? And why the calculation were global in the multivariable step ?<br>The Type 3 Analysis of Effects for each variable should be added in both univariable and multivariable regressions.
